# Metabolic syndrome and its components are associated with hypoxemia after surgery for acute type A aortic dissection: an observational study

**DOI:** 10.1186/s13019-022-01901-y

**Published:** 2022-06-13

**Authors:** Like Zhang, Lei Zhang, Zengren Zhao, Yun Liu, Juzeng Wang, Mengye Niu, Xiansheng Sun, Xiansheng Zhao

**Affiliations:** Department of Vascular Surgery, The First Hospital of Hebei Medical University, No. 89 Donggang Street, Yuhua District, Shijiazhuang, 500000 Hebei China

**Keywords:** Acute type A aortic dissection, Metabolic syndrome, Hypoxemia, Components, Scoring system

## Abstract

**Background:**

The aim of this study was to explore whether or to what extent metabolic syndrome (METs) and its components were associated with hypoxemia in acute type A aortic dissection (ATAAD) patients after surgery.

**Methods:**

This study involved 271 inpatients who underwent surgery. Demographic and clinical data were collected. Subgroup analysis, mixed model regression analysis, and receiver operating characteristic (ROC) curve analysis were performed, and a scoring system was evaluated.

**Results:**

The 271 inpatients were assigned to the hypoxemia group (n = 48) or no hypoxemia group (n = 223) regardless of METs status. Compared to the no hypoxemia group, the hypoxemia group had a higher incidence of METs. Hypoxemia was present in 0%, 3.7%, 19.8%, 51.5%, 90.0% and 100% in the groups of individuals who met the diagnostic criteria of MetS 0, 1, 2, 3, 4 and 5 times, respectively. In the multivariable logistic regression analysis, BMI quartile was still a risk factor for hypoxemia after adjustment for other risk factors. After adjustment for potential confounding factors, METs was an independent risk factor for hypoxemia in several models. After assigning a score for each METs component present, the AUCs were 0.852 (95% CI 0.789–0.914) in all patients, 0.728 (95% CI 0.573–0.882) in patients with METs and 0.744 (95% CI 0.636–0.853) in patients without METs according to receiver operating characteristic analysis.

**Conclusions:**

METs, especially body mass index, confers a greater risk of hypoxemia in ATAAD after surgery.

## Background

Acute type A aortic dissection (ATAAD) is a life-threatening cardiovascular disease with high mortality; the mortality rate is approximately 27% after surgery and approximately 58% with noninvasive treatment [1]. Despite significant improvements in surgical techniques, postoperative mortality is still high for ATAAD due to the incidence of complications [2]. Hypoxemia is the most common symptom of acute lung injury and is characterized by a ratio of arterial partial pressure of oxygen to fraction of inspired oxygen (PaO_2_/FiO_2_) ≤ 300 mmHg, which also leads to increased mortality [3]. The underlying mechanisms of hypoxemia in ATAAD remain elusive. Previous studies found that systolic blood pressure levels, body mass index (BMI), and obesity were important indicators of the prognosis of hypoxemia in ATAAD [4–6]. In addition, glucose and hyperlipidemia are associated with hypoxemia in other systems [7, 8]. Metabolic syndrome (METs) is characterized by a cluster of risk components, including abdominal obesity, hyperglycemia, dyslipidemia and hypertension [9]. In general, METs affect post-operative care in nearly all other surgery, which was closely associated with the incidence of wound infections and surgical adhesions [10, 11] 30497091/26109210. For cardiovascular system, METs seems to be an independent predisposing factor for mortality after coronary artery bypass grafting surgery [12]. In valves surgery, METs is a tendency to accelerated development of a pressure gradient and associate with the progression of aortic bioprosthetic valve stenosis [13]. However, the number of studies was relatively small about the relationship between metabolic syndrome and aortic dissection. In the present study, we investigated the association of METs and its components with the incidence of hypoxemia and determined the usefulness of METs for diagnosis of and risk assessment in ATAAD in clinical practice, providing new insight into the incidence of hypoxemia.

## Methods

### Study cohort

**Fig. 1:**
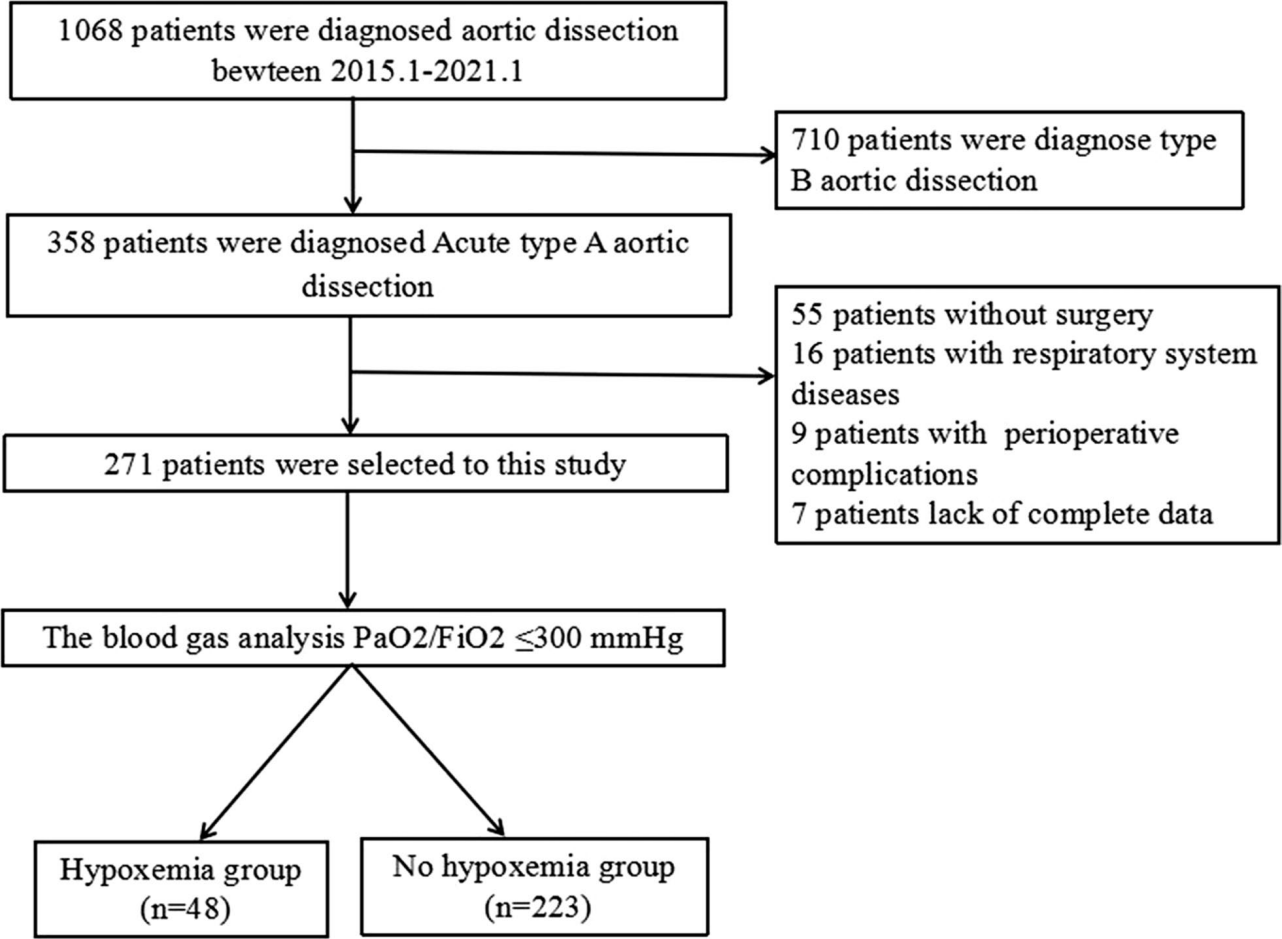
Population flowchart of enrolled patients

This is an observational and retrospective study. A total of 271 consecutive ATAAD patients who received treatment in the Department of General Surgery at the First Hospital of Hebei Medical University were enrolled in this study from January 2015 to January 2021. The inclusion criteria were as follows: (1) diagnosed with ATAAD confirmed by CT angiography of the aorta and (2) underwent surgical treatment. The major exclusion criteria included the following: (1) patients with respiratory system diseases; (2) patients who did not undergo surgery; and (3) patients who suffered from any perioperative complications. According to arterial blood gas analysis, patients with PaO_2_/FiO_2_ ≤ 300 mmHg for the first 2 days after the operation were included in the hypoxemia group. Patients with PaO2/FiO2 greater than 300 mmHg formed the no hypoxemia group. The study was approved by the Institutional Review Board of the First Hospital of Hebei Medical University. All subjects provided written informed consent. The detailed recruitment process is shown in Fig. 1.

### Metabolic syndrome

According to the criteria of the American National Cholesterol Education Program [14], MetS was defined as the presence of three or more of the following criteria: body mass index (BMI) > 30 kg/m^2^, high-density lipoprotein (HDL) < 50 mg/dL among women and < 40 mg/dL among men, fasting plasma triglycerides (TG) ≥ 150 mg/dL, systolic blood pressure (SBP) ≥ 130 mmHg, diastolic blood pressure (DBP) ≥ 85 mmHg, fasting plasma glucose (FPG) ≥ 100 mg/dL or previously diagnosed type 2 diabetes mellitus (T2DM).

### Baseline demographic and clinical characteristics

Data regarding sex, age, body mass index (BMI), blood pressure, heart rate (HR), systolic blood pressure (SBP), diastolic blood pressure (DBP), ventricular ejection fraction (LVEF), history of hypertension (HT), type 2 diabetes mellitus (T2DM), coronary artery disease (CAD) and thoracic surgery were collected. Preoperative laboratory tests were performed within 24 h before surgery, including tests for fasting blood glucose (FBG), triglycerides (TG), high-density lipoprotein cholesterol (HDL-C), white blood cells (WBCs), platelets (PLTs), creatinine (Cr), uric acid (UA), troponin I, and red blood cells (RBCs). Surgery variables included length of surgery, cardiopulmonary bypass time, cross-clamp time, circulatory arrest, minimum temperature, ICU stay time, hospital stay time, PaO2/FiO2 and mechanical ventilation time.

### Statistical methods

Statistical computations were performed using SPSS v24.0 (IBM Inc., Armonk, NY, USA). Continuous variables are reported as the mean ± standard deviation for normally distributed data or as the median and quartiles (Q1, Q3) for nonnormally distributed data. Discrete variables are expressed as frequencies and percentages and were compared using the chi-square test. Multivariable logistic regression analyses were performed to detect the relationship between hypoxemia and METs. In the multivariate analysis, odds ratios (ORs) and 95% confidence intervals (CIs) for hypoxemia were calculated using a logistic regression model after adjusting for potential confounding variables. To verify the robustness of our results, subgroup analyses were performed to explore the association between the number of MetS components and hypoxemia. These predictors of metabolic syndrome components were assigned points based on their regression coefficient, and a scoring system was produced. Receiver operating characteristic (ROC) curves were constructed, and the areas under the curves (AUCs) were calculated to assess the discriminatory power of the scoring system for MetS. A two-sided *p* value < 0.05 was considered statistically significant.

## Results

### Baseline demographic and clinical characteristics

Table 1 summarizes the clinical characteristics of the hypoxemia group (n = 48) and no hypoxemia group (n = 223). The mean age was 53.4 ± 7.2 and 52.9 ± 6.2 years in the two groups, respectively. The incidence of aortic valve disease, Marfan syndrome, CAD, and history of thoracic surgery were not significantly different between the groups (all *p* > 0.05). Compared to the control group, the hypoxemia group had significantly greater BMI, SBP, TG and WBC values and a longer length of surgery, ICU stay time, hospital stay time and mechanical ventilation time, and the differences were statistically significant (all *p* < 0.05). Similarly, there were statistically significant differences in the incidence of HT, T2DM, metabolic syndrome and smoking (all *p* > 0.05).

**Table1:** Clinical characteristics in two group

Variables	Hypoxemia group	No hypoxemia group	*x*^2^/*t*	*p* value
	n = 48	n = 223		
Male, n (%)	37 (77.1)	164 (73.5)	0.258	0.611
Age, years	53.4 ± 7.2	52.9 ± 6.2	0.555	0.579
BMI, Kg/m^2^	29.2 ± 4.1	25.8 ± 2.7	− 7.277	< 0.001
HT, n (%)	32 (66.7)	113 (50.7)	4.062	0.044
T2DM, n (%)	19 (39.6)	26 (11.7)	22.241	< 0.001
Aortic valve disease, n (%)	4 (8.3)	16 (7.2)	0.078	0.781
Metabolic syndrome, n (%)	27 (56.3)	17 (7.6)	68.673	< 0.001
Smoker, n (%)	31 (64.6)	96 (43.0)	7.355	0.007
Marfan syndrome, n (%)	1 (2.1)	1 (0.4)	1.441	0.230
CAD, n (%)	7 (14.6)	34 (15.2)	0.014	0.907
History of thoracic surgery, n (%)	2(4.2)	21 (9.4)	1.402	0.236
SBP, mmHg	150.5 ± 13.4	143.4 ± 13.8	− 3.264	0.001
DBP, mmHg	80.3 ± 6.2	79.1 ± 6.7	− 1.110	0.268
HR, bpm	70.1 ± 12.3	71.7 ± 11.1	0.177	0.383
LVEF, %	56.9 ± 6.0	57.4 ± 6.5	0.480	0.632
Troponin I, ng/mL	0.01 (0, 0.01)	0 (0, 0.01)	− 1.103	0.270
FBG, mmol/L	5.0 ± 0.7	4.9 ± 0.4	− 1.229	0.220
TG, mmol/L	1.5 (1.1, 2.6)	1.2 (1.0, 1.5)	− 2.620	0.009
HDL-C, mmol/L	1.2 (0.9, 1.8)	1.6 (1.1, 2.2)	− 3.925	< 0.001
WBC,10^12^/L	11.4 ± 1.9	10.0 ± 1.4	− 5.961	< 0.001
PLT, 10^9^/L	235.5 ± 67.8	217.6 ± 58.9	− 1.653	0.100
RBC, 10^12^/L	4.6 ± 0.6	4.6 ± 0.5	− 0.426	0.670
Cr, μmol/L	75.4 ± 16.4	76.2 ± 21.3	0.246	0.806
UA, μmol/L	349.0 ± 91.9	338.6 ± 76.9	0.725	0.469
eGFR, mL/(min·1.73 m2)	90.9 ± 14.3	90.5 ± 16.2	− 0.145	0.885
Length of surgery, min	287.3 ± 19.4	276.2 ± 24.9	− 2.913	0.004
Cardiopulmonary bypass time, min	167.5 ± 22.5	170.0 ± 23.0	0.674	0.501
Cross-clamp time, min	87.7 ± 13.6	88.7 ± 13.0	0.465	0.642
Circulatory arrest, min	43.4 ± 7.3	42.1 ± 7.5	− 1.076	0.283
Minimum temperature, °C	26.0 ± 0.5	26.0 ± 0.5	1.105	0.270
ICU stay time, day	6.7 ± 1.6	5.4 ± 1.5	− 5.488	< 0.001
Hospital stay time, day	20.0 ± 3.7	17.1 ± 4.2	− 4.522	< 0.001
PaO2/FiO2, mmHg	256.4 ± 24.4	330.8 ± 13.5	29.346	< 0.001
Mechanical ventilation time, hour	39.2 ± 21.9	21.1 ± 8.2	− 9.587	< 0.001
Elevated BMI, n (%)	18 (37.5)	11 (4.9)	43.839	< 0.001
Elevated BP, n (%)	47 (97.9)	203 (91.0)	2.619	0.106
Elevated FBG, n (%)	23 (47.9)	31 (13.9)	28.643	< 0.001
Reduced HDL-C, n (%)	25 (52.1)	42 (18.8)	23.462	< 0.001
Elevated TG, n (%)	16 (33.3)	26 (11.7)	14.168	< 0.001

BMI, body mass index; HT, Hypertension; T2DM, type 2 diabetes mellitus; SBP, systolic blood pressure; DBP, diastolic blood pressure; HR, heart rate; FBG, Fasting blood glucose; TG, Triglycerides; HDL-C, high-density lipoprotein cholesterol; WBC, white blood cell; PLT, Platelet; Cr, creatinine; UA, Uric acid; LVEF, ventricular ejection fraction

### METs incidence and clinical characteristics

**Fig. 2:**
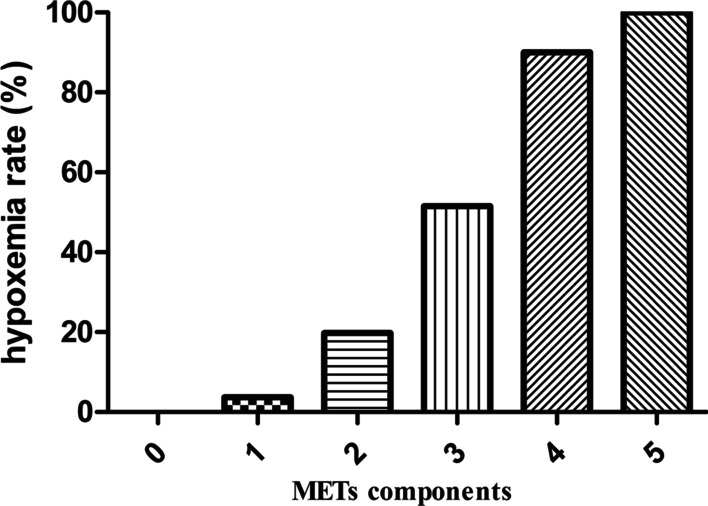
Hypoxemia rate of metabolic syndrome components

Participants were divided into six groups according to whether they met 0, 1, 2, 3, 4 or 5 of the METs diagnostic criteria, and there were 10 (3.7%), 136 (50.2%), 81 (29.9%), 33 (12.2%), 10 (3.7%) and 1 (0.3%) individuals in the respective groups. Hypoxemia was present in 0%, 3.7%, 19.8%, 51.5%, 90.0% and 100% of the six groups, with significant differences among groups (*p* < 0.05, Table 2 Fig. 2). The prevalence of males was the highest in the 0 group. There were significant differences among the six groups in terms of mechanical ventilation time, WBC, PaO2/FiO2, cross-clamp time and LVEF (all *p* < 0.001). The MetS components BMI, HT, T2DM, SBP, and TG increased with increasing numbers of traits. For comparisons among groups, the greatest difference was in BMI. (*p* < 0001, Table 2).

**Table 2:** Baseline characteristics of the number of METs

Variables	The number of the presence of METs							
	0	1	2	3	4	5	*p* value	*p* < 0.05
	n = 10	n = 136	n = 81	n = 33	n = 10	n = 1		
Male, n (%)	8 (80.0)	101 (74.3)	62 (76.5)	21 (63.6)	8 (80.0)	1 (100.0)	0.723	c,e,i,l
Age, years	52.1 ± 3.9	53.1 ± 6.5	53.0 ± 5.6	52.3 ± 7.7	52.9 ± 7.4	65	0.521	e,l
BMI, Kg/m^2^	24.3 ± 1.7	25.4 ± 2.3	26.9 ± 2.9	27.2 ± 3.4	33.7 ± 5.0	35.0	< 0.001	b,c,d,e,f,g,h,i,k,l,m,n
HT, n (%)	0 (0)	71 (52.5)	45 (55.6)	23 (69.7)	5 (50.0)	1 (100.0)	0.006	a,b,c,d,e,i,l,n,o
T2DM, n (%)	0 (0)	3 (2.2)	18 (22.2)	18 (54.5)	5 (50.0)	1 (100.0)	< 0.001	b,c,d,e,g,h,i,l,m,n,o
Hypoxemia, n (%)	0 (0)	5 (3.7)	16 (19.8)	17 (51.5)	9 (90.0)	1 (100.0)	< 0.001	b,c,d,e,g,h,i,j,k,l,m,n,o
SBP, mmHg	123.8 ± 6.5	144.8 ± 14.8	144.7 ± 11.7	148.9 ± 13.0	149.7 ± 9.4	149	< 0.001	a,b,c,d,e
DBP, mmHg	72.2 ± 6.7	79.5 ± 6.4	79.3 ± 6.8	80.4 ± 6.5	81.5 ± 6.6	80	0.019	a,b,c,d
HR, bpm	68.8 ± 5.3	73.3 ± 12.9	70.5 ± 9.3	69.0 ± 7.9	65.2 ± 12.9	59	0.063	
FBG, mmol/L	5.2 ± 0.3	4.9 ± 0.4	5.0 ± 0.5	5.1 ± 0.6	5.1 ± 0.8	6.3	0.002	a,e,f,g,i,l,n
TG, mmol/L	1.2 (0.9, 1.3)	1.2 (1.0, 1.4)	1.3 (1.1, 1.6)	1.5 (1.2, 2.1)	2.7 (2.1, 3.3)	2.4	< 0.001	c,d,e,f,g,h,i,k,m
Mechanical ventilation time, hour	19.9 ± 7.9	21.6 ± 9.2	25.2 ± 13.4	29.9 ± 12.8	40.1 ± 18.8	36	< 0.001	d,f,g,h,k
WBC,10^12^/L	9.6 ± 2.0	10.1 ± 1.4	10.0 ± 1.7	10.6 ± 1.5	11.6 ± 2.2	10.3	0.024	d,h,k
PLT, 10^9^/L	198.5 ± 32.7	217.1 ± 56.0	225.5 ± 63.0	225.0 ± 70.4	215.8 ± 77.2	372	0.118	e,i,l
PaO_2_/FiO_2_, mmHg	334.9 ± 12.5	328.2 ± 15.9	321.2 ± 28.2	285.4 ± 43.8	243.8 ± 33.8	224	< 0.001	d,e,f,g,h,i,j,k,l,m
Length of surgery, min	293.1 ± 30.7	275.1 ± 24.9	279.2 ± 23.5	281.0 ± 22.3	284.8 ± 18.8	298	0.163	a
Cardiopulmonary bypass time, min	164.4 ± 16.7	167.5 ± 21.9	172.8 ± 24.3	167.4 ± 24.1	183.2 ± 23.6	163.0	0.212	h
Cross-clamp time, min	87.9 ± 10.7	88.8 ± 13.3	89.4 ± 12.6	82.8 ± 11.6	95.9 ± 17.0	103	0.049	g,j,m
Circulatory arrest, min	45.6 ± 5.8	42.2 ± 6.9	42.2 ± 8.3	41.3 ± 6.2	46.5 ± 12.1	40.6	0.333	
Minimum temperature	25.9 ± 0.5	26.1 ± 0.5	26.0 ± 0.5	26.0 ± 0.5	26.2 ± 0.5	25.2	0.312	
HDL-C, mmol/L	1.5 (1.2, 2.2)	1.8 (1.3, 2.3)	1.3 (0.9, 1.8)	1.0 (0.9, 1.5)	0.9 (0.8, 1.5)	0.8	< 0.001	c,f,g,h,j
LVEF, %	60.5 ± 7.6	57.3 ± 6.2	58.0 ± 6.3	54.7 ± 6.2	57.7 ± 7.6	58	0.100	c,g,j

a, 0vs1; b, 0vs2; c, 0vs3; d, 0vs4; e, 0vs5; f, 1vs2; g, 1vs3; h, 1vs4; i, 1vs5; g, 2vs3; k, 2vs4; l, 2vs5; m, 3vs4; n, 3vs5; o, 4vs5

METs, Metabolic Syndrome; BMI, body mass index; HT, Hypertension; T2DM, type 2 diabetes mellitus; SBP, systolic blood pressure; DBP, diastolic blood pressure; HR, heart rate; FBG, Fasting blood glucose; TG, Triglycerides; HDL-C, high-density lipoprotein cholesterol; LVEF, ventricular ejection fraction

### METs components and hypoxemia

After adjustment for some potential risk factors, such as age, male sex, HR, CAD, previous thoracic surgery, and smoking, BMI quartiles (adjusted OR = 2.616, 95% CI 1.743–3.924, *p* < 0.001), HDL (adjusted OR = 0.560, 95% CI 0.393–0.799, *p* < 0.001) and SBP (adjusted OR = 1.646, 95% CI 1.145–2.367, *P* = 0.007) remained independent factors of hypoxemia. People with T2DM had a significantly increased risk of hypoxemia compared with those with no T2DM in all groups (adjusted OR = 5.460, 95% CI 2.211–13.484, *p* < 0.001). Compared with the first BMI quartile, the second, third and fourth BMI quartiles had ORs of incident hypoxemia of 6.124 (95% CI 1.056–35.493), 6.269 (95% CI 1.142–34.409), and 33.918 (95% CI 6.084–189.102), respectively, after adjusting for potential risk factors (Table 3).

**Table 3:** Impact of MetS components on patients with hypoxemia

Variables	Quartiles of components		All		
	Range	n	Hypoxemia/No hypoxemia	OR (95% CI)	*p* Value
HT, n (%)	–	145	32/113	1.827 (0.820–4.067)	0.140
T2DM, n (%)	–	45	19/26	5.460 (2.211–13.484)	< 0.001
BMI, Kg/m^2^	Per quartile	271	48/223	2.616 (1.743–3.924)	< 0.001
	Q1 ≤ 24.44	68	2/66	–	–
	24.22 < Q2 ≤ 26.02	68	8/60	6.124 (1.056–35.493)	0.043
	26.02 < Q3 ≤ 27.88	68	10/58	6.269 (1.142–34.409)	0.035
	27.88 < Q4	67	28/39	33.918 (6.084–189.102)	< 0.001
TG, mmol/L	Per quartile	271	48/223	1.236 (0.870–1.756)	0.238
	Q1 ≤ 1.04	70	11/59	–	–
	1.04 < Q2 ≤ 1.23	66	9/57	1.370 (0.411–4.569)	0.608
	1.23 < Q3 ≤ 1.49	67	5/62	0.261 (0.062–1.091)	0.066
	1.69 < Q4	68	23/45	2.268 (0.696–7.384)	0.174
HDL-C, mmol/L	Per quartile	271	48/223	0.560 (0.393–0.799)	0.001
	Q1 ≤ 1.15	65	23/42	–	–
	1.15 < Q2 ≤ 1.56	71	9/62	0.517 (0.157–1.708)	0.279
	1.56 < Q3 ≤ 2.12	68	10/58	0.383 (0.126–1.167)	0.091
	2.12 < Q4	67	6/61	0.091 (0.024–0.351)	< 0.001
SBP, mmHg	Per quartile	271	48/223	1.646 (1.145–2.367)	0.007
	Q1 ≤ 135	66	5/61	–	–
	135 < Q2 ≤ 143	70	11/59	4.178 (0.901–19.367)	0.068
	143 < Q3 ≤ 153	68	13/55	3.265 (0.737–14.462)	0.119
	153 < Q4	67	19/48	7.367 (1.676–32.377)	0.008
DBP, mmHg	Per quartile	271	48/223	0.988 (0.678–1.441)	0.952
	Q1 ≤ 75	70	10/60	–	
	75^<^Q2 ≤ 80	69	10/59	0.606 (0.153–2.399)	0.475
	80 < Q3 ≤ 84	62	16/46	1.148 (0.351–4.180)	0.834
	84^<^Q4	70	12/58	0.557 (0.142–2.182)	0.401
FBG, mmol/L	Per quartile	271	48/223	0.976 (0.698–1.366)	0.889
	Q1 ≤ 4.58	66	12/54	–	
	4.62 < Q2 ≤ 4.93	68	13/55	0.989 (0.312–3.141)	0.986
	4.93 < Q3 ≤ 5.29	69	6/63	0.353 (0.082–1.522)	0.163
	5.29 < Q4	68	17/51	0.951 (0.303–2.987)	0.932

a Multiple adjustment for Age, Male, HR, CAD, Previous thoracic surgery, Aortic valve disease, Marfan syndrome, smoker, LVEF, Length of surgery, Cardiopulmonary bypass time, Cross-clamp time, Circulatory arrest, Minimum temperature, TroponinI, WBC, PLT, RBC, ICU stay time, Hospital stay time, Mechanical ventilation time, PaO2/FiO2, Cr, eGFR, UA

OR, odds ratio; CI, confidence interval; METs, Metabolic Syndrome; BMI, body mass index; HT, Hypertension; T2DM, type 2 diabetes mellitus; HDL-C, high-density lipoprotein cholesterol, TG, Triglycerides, FBG, Fasting blood glucose; SBP, systolic blood pressure; DBP, diastolic blood pressure

### METs and hypoxemia

Table 4 shows the results of multivariate logistic regression analysis of the association between the incidence of hypoxemia and MetS. There were five models that adjusted for age, male sex, HR, CAD, previous thoracic surgery, aortic valve disease, Marfan syndrome, smoking status, LVEF, length of surgery, cardiopulmonary bypass time, cross-clamp time, circulatory arrest, minimum temperature, troponin I, WBCs, PLTs, RBCs, ICU stay time, hospital stay time, mechanical ventilation time, PaO2/FiO2, Cr, eGFR, UA, elevated body mass index, elevated blood pressure, elevated fasting glucose, reduced high-density lipoprotein cholesterol, and elevated triglycerides. The ORs were 17.112, 20.521, 31.229, 40.132, and 68.053 for MetS in Models 1, 2, 3, 4, and 5, respectively (all *p* < 0.05).

**Table 4:** Odds ratio and 95% confidence interval for hypoxemia

Models	METs	
	*OR* (95%CI)	*p* Value
Model 1	17.112 (7.742–37.821)	< 0.001
Model 2	20.521 (8.921–47.203)	< 0.001
Model 3	31.229 (11.295–86.341)	< 0.001
Model 4	40.132 (5.461–294.906)	< 0.001
Model 5	68.053 (2.026–2283.417)	0.019

Model 1, adjusted for Age, Male, HR; Model 2, adjusted for Age, Male, HR, CAD, Previous thoracic surgey, Aortic valve disease, Marfan syndrome; Model 3, adjusted for Age, Male, HR, CAD, Previous thoracic surgery, Aortic valve disease, Marfan syndrome, smoker, LVEF, Length of surgery, Cardiopulmonary bypass time, Cross-clamp time, Circulatory arrest, Minimum temperature; Model 4, adjusted for Age, Male, HR, CAD, Previous thoracic surgery, Aortic valve disease, Marfan syndrome, smoker, LVEF, Length of surgery, Cardiopulmonary bypass time, Cross-clamp time, Circulatory arrest, Minimum temperature, TroponinI, WBC, PLT, RBC, ICU stay time, Hospital stay time, Mechanical ventilation time, PaO_2_/FiO_2_, Cr, eGFR, UA; Model 5, adjusted for Age, Male, HR, CAD, Previous thoracic surgery, Aortic valve disease, Marfan syndrome, smoker, LVEF, Length of surgery, Cardiopulmonary bypass time, Cross-clamp time, Circulatory arrest, Minimum temperature, TroponinI, WBC, PLT, RBC, ICU stay time, Hospital stay time, Mechanical ventilation time, PaO_2_/FiO_2_, Cr, eGFR, UA, elevated body mass index, elevated blood pressure, elevated fasting glucose, reduced high-density lipoprotein cholesterol, elevated triglycerides

### METs scoring system and ROC curve analysis

**Fig. 3:**
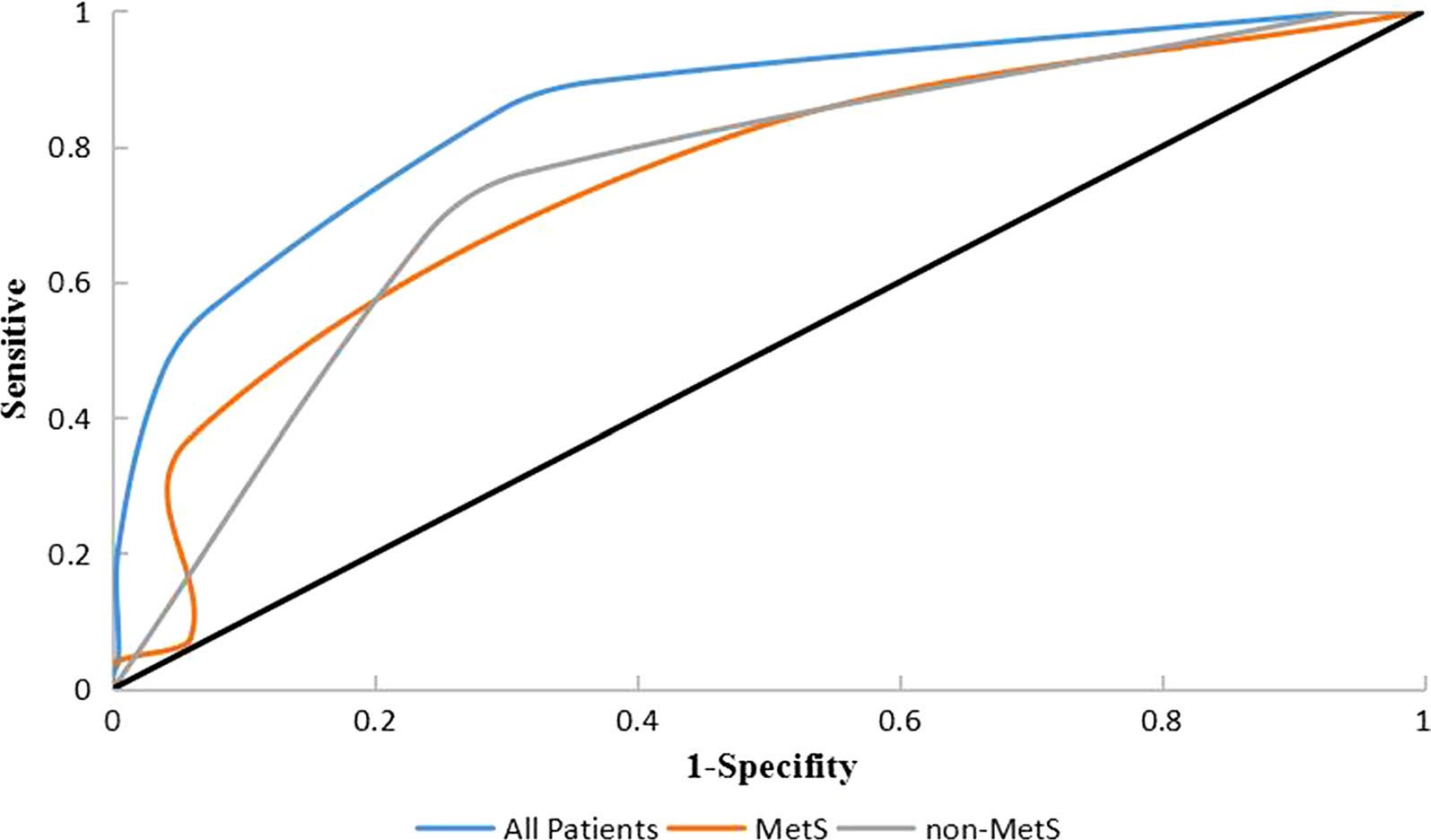
ROC curve analysis of the scoring system for the prediction of hypoxemia

Based on the regression coefficient, a point was assigned to each METs component. Elevated BMI was given 2 points, elevated BP was given 2 points, elevated FBG was given 1 point, reduced HDL was given 1 point, and elevated TG was given 1 point (Table 5). ROC curves were constructed to evaluate the scoring system. The AUCs were 0.852 (95% CI 0.789–0.914) in all patients, 0.728 (95% CI 0.573–0.882) in patients with METs and 0.744 (95% CI 0.636–0.853) in patients without METs (Table 6 Fig. 3).

**Table 5:** Multivariable analysis of the METs components

Variables	*OR* (95%CI)	*p* Value	Regression coefficient	Point
Elevated BMI	12.084 (4.193–34.828)	< 0.001	2.482	2
Elevated BP	9.829 (0.867–111.455)	0.065	2.285	2
Elevated FBG	5.814 (2.538–13.318)	< 0.001	1.760	1
Reduced HDL-C	5.300 (2.358–11.914)	< 0.001	1.668	1
Elevated TG	2.822 (1.100–7.238)	0.031	1.037	1

BMI, body mass index; BP, blood pressure; FBG, Fasting blood glucose; HDL-C, high-density lipoprotein cholesterol; TG, Triglycerides

**Table 6:** The ROC Curve analysis of the METs with hypoxemia

Factors	AUC	*P*	95% CI	Se (%)	Sp (%)	Cut off point
All patients	0.852	< *0.001*	*0.789–0.914*	*85.40*	*70.40*	4
MetS	0.728	*0.012*	*0.573–0.882*	*81.50*	*52.90*	6
non-MetS	0.744	< *0.001*	0.636–0.853	76.20	68.40	3

*P* < 0.05 was defined as statistically significant

Se, sensitive; SP, specifity

## Discussion

In this study, we demonstrated that METs components could significantly predict the incidence of hypoxemia in ATAAD patients according to multivariable and subgroup analyses. After adjusting for confounding factors, METs was also an independent risk factor for hypoxemia. Among METs components, BMI was the strongest predictor of hypoxemia, and the scoring system showed good predictive power.

As a result of high blood pressure, the aortic intima tears and progressive separation of the aortic wall layers results in the formation of a false lumen; this involves the ascending aorta and is classified as ATAAD [15]. With the advancement of surgery and postoperative management, the mortality of ATAAD has decreased significantly. Perioperative complications, including hepatic dysfunction, acute renal failure and neurological complications, are the main cause of death in ATAAD patients [16]. The occurrence of hypoxemia, a common complication, reaches 51% after surgery, which may lead to acute lung injury and influence recovery from the disease [17]. The main underlying mechanisms of hypoxemia after surgery in ATAAD remain unclear. Previous studies found that hypoxemia may be associated with an imbalance in ventilation and perfusion during acute bleeding [18]. In addition, inflammatory reactions and oxidative stress play an important role, damaging alveolar epithelial and capillary endothelial cells [4]. Ming Gong et al. enrolled 112 consecutive ATAAD patients who underwent surgery. They found that BMI (OR = 1.473) and female sex (OR = 12.978) were independent risk factors for hypoxemia after multivariate logistic regression analysis [19]. Recently, a cohort study with 172 ATAAD patients explored inflammation biomarkers, such as interleukin-6 and C-reactive protein, associated with the incidence of preoperative hypoxemia [5].

METs comprises five components, BMI, blood pressure, fasting plasma glucose, high-density lipoprotein and triglycerides, which are often ignored in clinic practice [20]. It also represents a cluster of metabolic abnormalities that reflect changes in human physical performance, such as insulin resistance and neurohormonal activation. In the final common pathway, a series of inflammation signaling cascades are triggered, leading to clinical manifestations [21]. We found that METs was robustly associated with hypoxemia in logistic and subgroup analyses, especially with respect to BMI. The present study provides new insight for clinical practice in that METs may indicate an inflammatory state in the body and should be given more attention. It also indicates that the pathogenesis of hypoxemia may be a multifactorial process. Inflammation, insulin resistance and lipid abnormalities exert synergistic antitumor effects on the process of hypoxemia.

Several studies have found that BMI is an independent risk factor for hypoxemia [5, 6]. Abundant amounts of cytokines and reactive oxygen species are released from adipose tissue in obesity, which leads to abnormal ventilation perfusion and decreased pulmonary gas exchange [22]. Previous studies have shown that hypoxemia is associated with decreased insulin sensitivity and varying degrees of insulin resistance [23]. At high blood glucose levels, oxygen transport and carbon monoxide diffusing capacity are decreased in the lungs [24]. In ATAAD patients, the incidence of T2DM was higher in the severe hypoxemia group than in the nonsevere hypoxemia group (12.1% *vs.* 1.4%, *P* = 0.05) [19]. There is still some controversy about the relationship between blood pressure and hypoxemia. Guo Z et al. found that systolic blood pressure was a protective factor against preoperative hypoxemia in ATAAD patients [4]. However, systolic blood pressure was higher in the hypoxemia group. Blood pressure is reflective of the sympathetic state and systemic vascular resistance [25]. When the renin-angiotensin system induces inflammatory cascades, alveolar capillary membrane permeability and pulmonary vascular resistance are increased, leading to hypoxemia after surgery [26]. Lipids, an important factor in METs, play an important role in the modulation of inflammation. In addition, the hypoxia-inducible factor 1-vascular endothelial growth factor pathway is important in hypoxia and is regulated by high-density lipoproteins [27]. Triglyceride levels are also associated with hypoxia-inducible lipid droplet-associated protein and hypoxia inducible gene-2, which involve the process of hypoxemia [28]. In our study, reduced HDL-C and elevated TG levels were independent risk factors for hypoxemia. In the future, large studies need to be conducted to confirm the role of high-density lipoproteins and triglycerides in the development of hypoxemia.

To date, several METs diagnostic criteria, including the National Cholesterol Education Program (NCEP), International Diabetes Federation (IDF), and American Heart Association/National Heart, Lung, and Blood Institute (AHA/NHLBI) criteria, have been proposed. Considering cardiovascular disease, the NCEP METs definition may be more suitable in the Chinese population. Compared to the AHA/NHLBI and IDF criteria, the NCEP criteria better detect the prevalence of cardiovascular disease (OR: 1.40) [29]. Therefore, we chose the NCEP criteria seemed to be more suitable for our study.

## Limitations

Our study has some limitations. First, this was an observational study with a single center and a small number of enrolled patients, which may have introduced selection bias. In future, enlarging the sample size and randomized controlled trials need to be performed to confirm our results. Second, the underlying mechanistic link between MetS and hypoxemia is not clear, and unidentified risk factors may affect the incidence of hypoxemia. Animal model studies and prospective clinical observations will perform to explore deeper relationship. Furthermore, we focus on the the occurrence of hypoxemia after surgery and explore the prognosis value of METs. In this study, we not explore if interventions can reduce morbidity and mortality after hypoxemia. In further,Long-term follow up is necessary and find the influences of different treatments on the outcome.

## Conclusions

For ATAAD patients, the occurrence of hypoxemia after surgery seems tightly linked to METs, especially BMI. After adjusting for potential risk factors and establishing a scoring system, METs was an independent risk factor for hypoxemia. Our research indicates that hypoxemia may be a multifactorial process and that endocrine disorders that activate systemic inflammation may play an important role in ATAAD patients after surgery.

## Data Availability

Data available on request from the authors.
